# Feasibility and Efficacy of Commercial-Off-the-Shelf Virtual Reality Applications for Managing Chronic Pain and Enhancing Well-Being Among Older Adults in the Community: Mixed Methods Pilot Study

**DOI:** 10.2196/67765

**Published:** 2025-08-18

**Authors:** Rachel Yim Fong Leung, Megan Zichen Ye, Flora Yaqian Zhang, Tyrone Tai-On Kwok, Yuying Sun, Agnes Yuen Kwan Lai, Juming Jiang, Mimi Mun Yee Tse

**Affiliations:** 1 School of Nursing and Health Sciences, Hong Kong Metropolitan University, 11th Floor, Jockey Club Institute of Healthcare, 1 Sheung Shing Street, Homantin, Kowloon, Hong Kong, 852, China, 852 39702944

**Keywords:** virtual reality, VR, commercial VR, off-the-shelf, non-localized, pain, older adults, well-being, NPRS, pain self-efficacy, WHO-5, mood, feasibility, pilot study, World Health Organization-Five Well-Being Index

## Abstract

**Background:**

Older adults may experience chronic pain as they age, which can affect their physical and psychological well-being. Virtual reality (VR) is emerging as a novel and nonpharmacological intervention that offers pain relief and mood enhancement through immersive experiences. However, the feasibility and effectiveness of using nonlocalized and commercial VR applications for chronic pain relief and mood enhancement among community-dwelling older adults remain underexplored.

**Objective:**

The main objectives of this study were to (1) evaluate the feasibility of using commercial-off-the-shelf VR applications for managing chronic pain among older adults, (2) assess the efficacy of VR in alleviating chronic pain, and (3) examine its impact on the well-being of older adults in a community setting.

**Methods:**

The study was a single-arm mixed methods pilot study. It was divided into two stages, including preparation and implementation. A total of 13 older adults (8 with chronic pain and 5 without) were recruited to participate in a 3-week VR intervention. Participants engaged in VR sessions that followed a step-by-step adaptation process. Each session included 360-degree relaxation videos and VR-based boxing exercises via Les Mills BODYCOMBAT, lasting 15 minutes. Pain intensity, pain self-efficacy, well-being, and mood were measured pre- and post-intervention using the Numeric Pain Rating Scale (NPRS), a pain self-efficacy question, the World Health Organization–Five Well-Being Index (WHO-5), and the Mood Assessment Scale (Mood). VR feasibility was evaluated based on completion rates, adverse outcomes, and qualitative feedback from semistructured interviews.

**Results:**

Of the 13 participants recruited, 11 completed the intervention (84.6% completion rate). The mean age was 79.2 (SD 9.2) years. The study found a statistically significant 16.32% improvement in the mean mood score, with a mean increase of 2.64 (SD 1.45) points and a large effect size (*P*<.001; Cohen *d*=1.82). The median pain self-efficacy score decreased from 3.0 (IQR 1.5-3.0) to 1.0 (IQR 1.0-2.0) (z=−2.236; *P*=.03). However, no significant changes were observed in pain intensity or overall well-being. The study demonstrated the high feasibility of commercial-off-the-shelf VR technology for older adults. Minor adverse effects were reported, including back pain and headset discomfort. In addition, 90.9% of participants enjoyed the VR experience, and all were willing to join future sessions.

**Conclusions:**

The pilot study demonstrated that commercial-off-the-shelf VR applications can effectively enhance mood and pain self-efficacy. Additional procedures, such as prebriefing, real-time interpretation, and a gradual adaptation process, were essential to overcoming barriers such as language, cultural nuances, and the digital literacy of older adults. Despite the lack of significant changes in pain intensity or overall well-being, the psychological benefits suggest that VR could be a valuable adjunct tool in chronic pain management. Future research should focus on larger sample sizes, longer intervention durations, randomized controlled trials, and the development of localized commercial VR applications to further explore their efficacy.

## Introduction

### Background

Since Ivan Sutherland and Bob Sproull created the first virtual reality (VR) headset in 1968, VR technology has evolved significantly [1]. VR involves experiencing digital simulations, ranging from realistic to fantastical, in 3D spaces using computer technology [2]. Users can experience VR in immersive and nonimmersive forms. Today, VR technology is widely used in gaming, entertainment, education, and therapy.

In the medical field, VR has gained recognition as a nonpharmacological tool for psychosocial and physical therapy. Research has consistently shown VR’s capacity to enhance mental health and cognitive abilities while diminishing symptoms of depression [3-6]. VR can assist in physical recovery and facilitate postsurgical rehabilitation in various clinical settings [7-9]. With the aging population and the cost of chronic pain potentially reaching up to US $635 billion annually [10], VR has also been developed to relieve persistent pain. Studies have validated its efficacy in pain reduction using techniques such as distraction, relaxation, graded exposure therapy, and pain management education [11-14]. Various VR applications have been designed and built specifically for chronic pain therapy. These include, but are not limited to, applications for lower back pain [15], neck pain [16,17], migraine headaches [18], knee osteoarthritis [19], and regional pain [20-22]. These VR applications are often used under the guidance of health care professionals such as physiotherapists or occupational therapists [16-18,21,22].

On the other hand, as chronic pain can affect various body parts, different studies have indicated the potential benefits of commercial-off-the-shelf VR applications for individuals with persistent pain [23-26]. One study explored the efficacy of using 12 commercial VR applications to provide veterans with chronic pain therapy, ranging from low-intensity distraction to high-intensity movement-based exposure. Benefits included reducing kinesiophobia, improving patient-specific functioning, and delivering pain distraction [23]. Another study aimed to desensitize a patient to visually evoked pain by using the Vacation Simulator (Owlchemy Labs), a commercial VR application. The results showed that the intervention could nearly extinguish the visually evoked pain by the end of the program [25]. Commercial VR applications may benefit individuals with chronic or multiple pain conditions, particularly those needing regular physical exercise [26-28]. Immersive commercial VR games can motivate people to work out to improve their health.

Locally, since 2005, the Hong Kong Hospital Authority has implemented VR for functional rehabilitation in walking, upper and lower limb movement, fall prevention, cognitive ability, and driving reaction, in which therapists supervise nonimmersive VR training to ensure patient safety [7]. However, research on VR’s efficacy for chronic pain remains limited. Two systematic reviews found VR to be effective in reducing chronic pain levels [29,30], but one study highlighted that its impact might depend on the methodology and strategies used in the research [29]. Moreover, 5 clinical trials in Hong Kong explored VR’s use in pain alleviation, but most trials primarily focused on acute pain relief in clinical settings such as peripheral intravenous cannulation [31], venipuncture [32], palliative care [33], and elective surgery [34]. Only one recent clinical trial demonstrated chronic pain reduction through VRiKnee [35], a designated VR application for knee osteoarthritis.

The challenges outlined above highlight the need to explore the use of commercial VR applications for older adults in the community in a local setting, without relying on high-cost, high-turnaround-time, tailor-made VR applications. However, the number of localized and commercial VR applications is limited. While international studies have explored commercial VR applications to manage chronic pain or well-being, many required participants to understand English. This often excluded older adults with limited English proficiency, as these applications were primarily in English. In addition, concerns regarding cultural nuances and perceived limited technological literacy were factors for exclusion in many studies [36-38]. Researchers often either specifically designed VR applications or used native-language and commercial-off-the-shelf VR applications for older adults in the community. There has been little research exploring the feasibility and effectiveness of applying nonlocalized, commercial VR applications to manage chronic pain and well-being in the population.

### Research Objectives

The main objectives of this study were to (1) evaluate the feasibility of using commercial-off-the-shelf VR applications for managing chronic pain among older adults, (2) assess the efficacy of VR in alleviating chronic pain, and (3) examine its impact on the well-being of older adults in a community setting.

## Methods

### Study Design

This study was a single-arm, mixed methods pilot study conducted between January and March 2024 at an elderly day care center in Hong Kong. The study was approved by the Ethical Committee of the Hong Kong Metropolitan University (reference no HE-RD/2023/1.15). Participants signed an informed consent form and completed a questionnaire that included demographic information, the Numeric Pain Rating Scale (NPRS), pain self-efficacy, medical history queries, and their VR experience for initial screening. The study comprised two major stages: preparation and implementation. This paper is reported under the CONSORT (Consolidated Standards of Reporting Trials) 2010 extension for pilot and feasibility studies (items 1a-12b, as applicable to a nonrandomized, single-arm design).

This exploratory feasibility pilot followed Julious’ [39] rule-of-thumb of 12 participants for pilot work. Accounting for possible attrition, we aimed for 14 enrollments and achieved 13 complete datasets. Using the large pre-post mood effect observed here (Cohen *d*=1.82), a conventional two-arm superiority trial would need roughly 34 participants in total (17 per arm) to provide 80 % power at *α*=.05, calculated with G*Power 3.1 (Heinrich Heine University Düsseldorf).

### Preparation Stage

This stage consisted of four key steps before the formal study: theory setting, game selection, recruitment, and a pre-pilot study.

#### Theories Setting

The study was grounded in 3 theoretical frameworks. The biopsychosocial model suggests that health and disease are influenced by biological, psychological, and social factors [40-42]. The commercial VR applications selected should positively impact all 3 dimensions. The second theory is the gate control theory of pain. It suggests that pain perception is both a direct result of injury and brain modulation. VR can provide relaxation and distraction, closing the gate to pain signals and reducing the pain experience [43,44]. The last framework is self-determination theory, which emphasizes the importance of autonomy, competence, and relatedness in motivating behavior [45]. The VR games selected should motivate older adults and enhance their well-being.

#### Game Selection

Two commercial VR applications were selected for the intervention: YouTube VR (Odders Lab) [46] and Les Mills BODYCOMBAT (Phillip Mills) [47]. The applications fulfilled specific criteria: supporting both standing and seated positions, offering adjustable difficulty levels, accommodating varying fitness levels, and featuring highly visual, intuitive content that minimized reliance on text or voice instructions. Many studies have used YouTube VR as a medium to manage patients with chronic pain in conditions such as cancer [31] and haemophilic ankle arthropathy [48]. These studies demonstrated the capabilities of immersive 360-degree videos to alleviate pain and improve well-being [33,48,49]. Videos were chosen based on 4K or higher resolution and ease of visualization, accommodating the visual requirements of older adults. The chosen videos included diverse themes (refer to Multimedia Appendix 1). As for VR boxing exercises from Les Mills BODYCOMBAT, it incorporates elements from martial arts to promote physical activity.

#### Recruitment

Participants were recruited from an elderly day care center in Hong Kong. The center provides day care services, rehabilitation exercises, and social activities for older adults aged 60 years or older, including those with cognitive impairment, who were assessed by the Standardized Care Need Assessment Mechanism for Elderly Services (SCNAMES). We included both pain and nonpain older adults to assess VR’s effects on general well-being, not just pain management. This also allowed us to compare the effectiveness and feasibility of VR between the two groups, making the findings more broadly applicable. The inclusion criteria were (1) aged 60 years or older, (2) residing in their own homes and not in a long-term care facility (such as a nursing home) within the last year, (3) willingness to participate in the VR intervention, (4) ability to understand Chinese and speak Cantonese, and (5) self-reported ability to see clearly for daily tasks (with habitual glasses if worn) and absence of diplopia. Participants were excluded if they had (1) an inability to communicate, (2) moderate to severe dementia in stage 4 or above under the Global Deterioration Scale (GDS), and (3) visual impairment or visual surgery within the past 1 year.

#### Prepilot Study

A prepilot study was conducted to evaluate the environment, process flow, and initial acceptance of VR by older adults. The prepilot also evaluated the eligibility of participants with visual impairments. Five older adults participated, revealing issues such as headset discomfort and latency in high-quality video playback. These issues were addressed by using a portable 5G router and refining written instructions to simplify VR usage. The feedback gathered helped refine the formal pilot study.

### Implementation Stage

#### Intervention

Eligible participants engaged in VR sessions once weekly for 3 consecutive weeks. Each session comprised two parts—a 10-minute YouTube VR immersive experience to introduce the VR environment and a 5-minute VR boxing exercise using Les Mills BODYCOMBAT for higher-intensity interaction. For safety, participants were seated during all sessions. The study used the Meta Quest 3, an immersive VR device with a head-mounted display and wireless handheld controllers. To ensure smooth adaptation to the digital environment, participants followed a sequential process of learning, familiarization, and interaction during each VR session, which was as follows:

##### Learning

**Figure 1.**
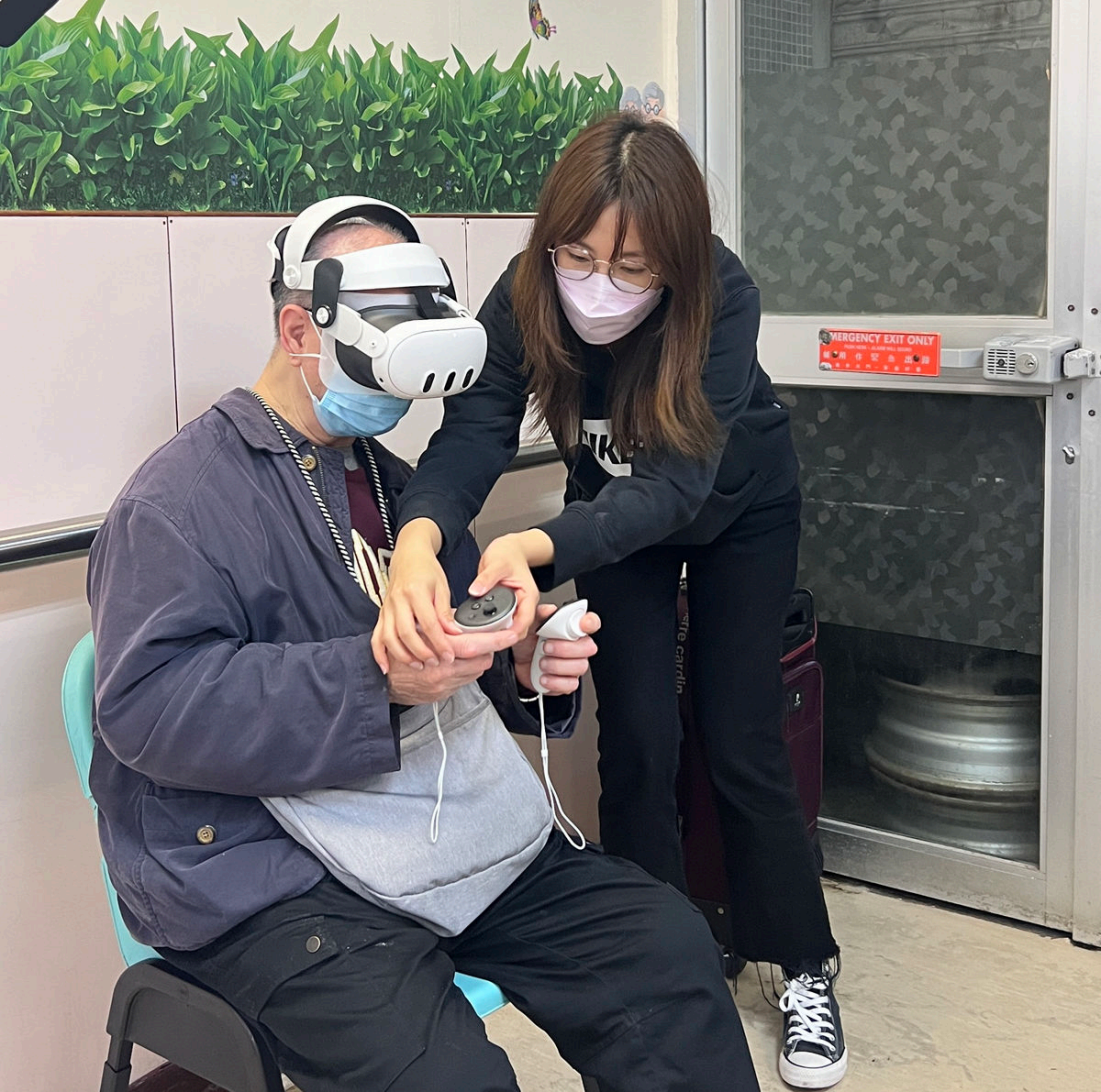
Guiding the participant to use the controllers.

Before each VR session, we conducted a 10-minute briefing with written instructions on how to use the controllers and what to expect during the session (Figures1  2).

Mood Assessment Scale (Mood) scores and potential discomfort were also assessed before starting. One-on-one assistance was provided throughout the intervention to help participants smoothly engage with the VR experience.

**Figure 2.**
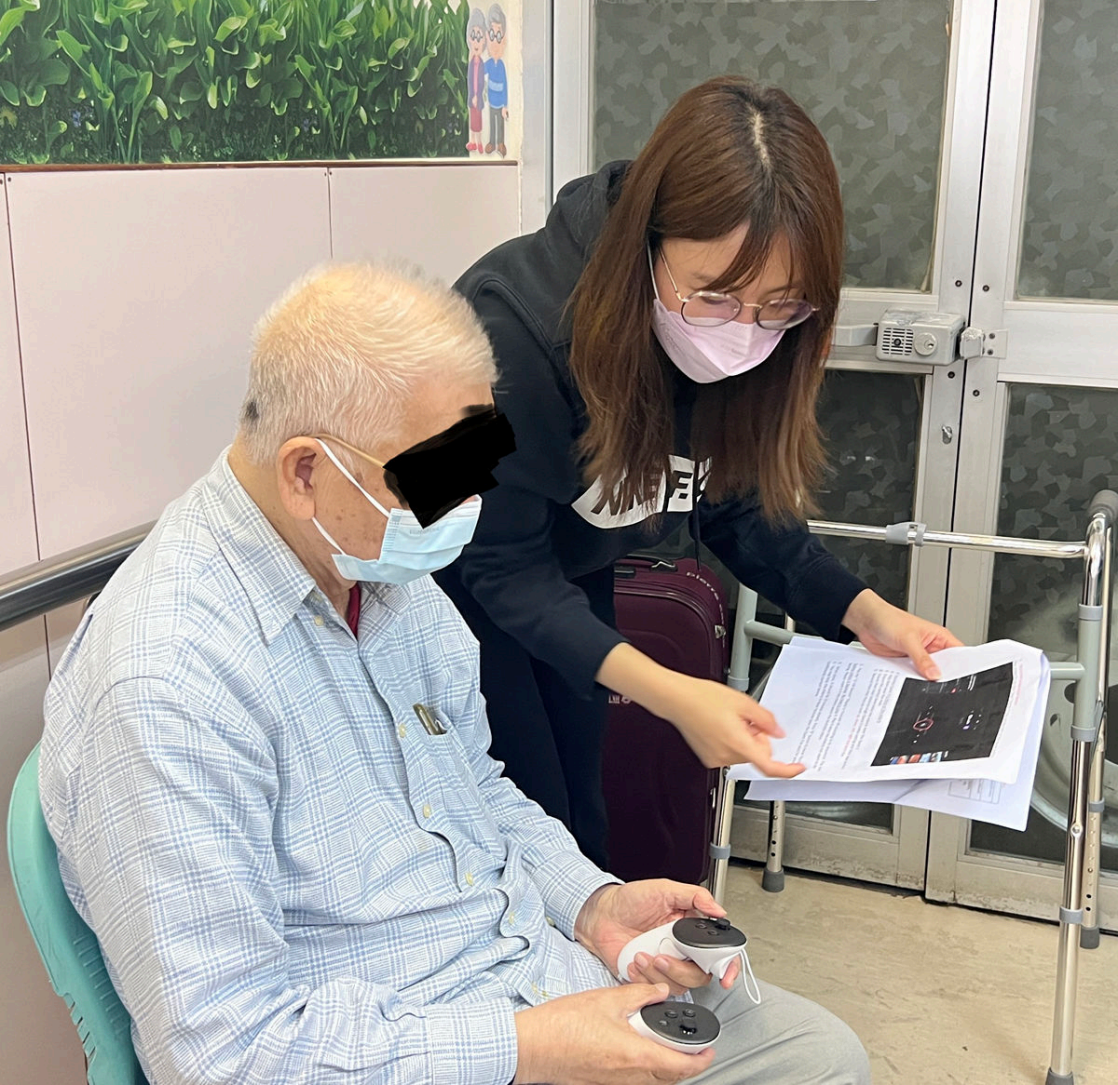
Individual briefing of the scenes to be seen.

##### Familiarization

Sessions began with YouTube VR to help participants acclimate to the VR environment. Real-time translation was provided for the English-language content. Videos typically lasted 3-5 minutes to maximize the participants’ VR experience.

##### Interaction

**Figure 3.**
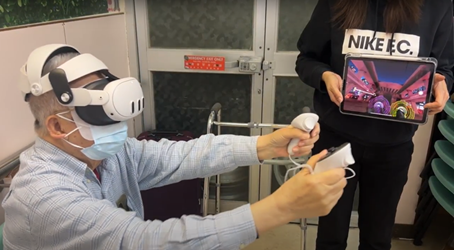
Participant engaging in Les Mills BODYCOMBAT, a 5-minute virtual reality boxing exercise.

After becoming familiar with the VR environment, the participants engaged in high movement-based activities in Les Mills BODYCOMBAT (Figures3  4). The VR classes, accessible in both standing and seated modes, included physical movements and cognitive challenges such as target hitting based on color and directional cues. Participants could begin with simpler, low-level classes and progress to more advanced levels involving punches and lateral movements for obstacle avoidance, provided they completed the lower levels successfully.

**Figure 4.**
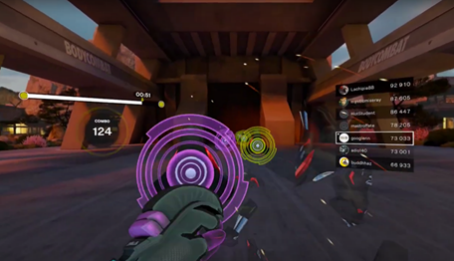
Scene from the Les Mills BODYCOMBAT game session.

### Data Collection

We gathered two categories of data: demographic and outcome data. Demographic data were collected at baseline when the potential participants had signed the consent form expressing their interest in joining the VR intervention.

The outcome data consisted of several measures: the World Health Organization–Five Well-Being Index (WHO-5), the NPRS, and assessments of pain self-efficacy, the Mood, and the feasibility of the VR intervention. In addition, we collected qualitative feedback from the participants after the last VR session.

To have a general picture of the effectiveness of VR in managing chronic pain and the well-being of the participants, the three outcome variables—NPRS, Pain Self-Efficacy, and WHO-5—were assessed before the first VR session (BT1) and after the end of the last VR session (AT3). To differentiate any potential change in mood before and after each VR intervention, the outcome variable “mood” was collected every time before (BT1, BT2, and BT3) and after each VR session (AT1, AT2, and AT3). In addition, the participants were checked to see if they had any discomfort immediately after every VR session. If the participants reported discomfort, they were assessed for VR sickness to understand the specific areas of discomfort.

#### The NPRS

The NPRS is a widely used tool in clinical and research settings to measure pain intensity. The NPRS uses a 0-10 scale, where 0 indicates “no pain” and 10 denotes “the most severe pain imaginable” [50]. The NPRS is generally considered reliable and has high reproducibility for measuring pain intensity across different patient populations and settings. Research has shown that the NPRS provides consistent results when used in repeated measures and correlates well with other pain scales such as the Visual Analog Scale (VAS) [51,52].

#### Pain Self-Efficacy

Pain self-efficacy was a belief held by people with chronic pain that certain activities can be carried out despite the pain [53]. In this pilot study, to minimize the cognitive load for older adults, we used a single question to examine the extent to which the pain affected their daily lives. Participants rated their responses to the item using a 5-point Likert scale, ranging from 0 (“none at all”) to 5 (“extremely”).

#### The WHO-5

The WHO-5 is a widely recognized and validated scale for use across many ethnicities, including the Hong Kong population [54-56]. There are 5 short and direct questions. All items are scored from 0 (“none of the time”) to 5 (“all the time”). Scores closer to the maximum reflect greater well-being. The raw score is obtained by summing the single response values (0‐5).

#### Mood Assessment Scale

There are four questions to capture the mood states of participants: (1) “I feel cheerful and in good spirits,” (2) “I feel relaxed,” (3) “I feel irritable,” and (4) “I feel scared.” The questions were inspired by the WHO-5 [54] and the Positive and Negative Affect Schedule (PANAS) [57]. Participants rated their current feelings using a 5-point Likert scale, with responses ranging from 1 (“not at all”) to 5 (“extremely”).

To calculate mood scores, the responses to the first two positive affect questions—feeling cheerful and relaxed—were scored from 1 to 5 points, directly corresponding to the intensity of the feeling. Conversely, responses to the negative affect questions—feeling irritable and scared—were inversely scored, with 5 points for “not at all” and 1 point for “extremely.” The scores for each session were summed and divided by the number of VR intervention sessions (3 in total) to calculate the average preintervention and postintervention mood scores. The methodology for calculating this composite mood score was tailored to accommodate the specific conditions and constraints of our study, including session count and participant demographics. It was also intended to minimize the possibility that outliers or particular incidents would disproportionately skew the results. Internal consistency of the 4-item scale in this sample was acceptable (Cronbach *α*=0.84, computed from 11 participants × 3 presession assessments, n=33).

#### Feasibility

The feasibility of the VR intervention was assessed through three primary measures: completion rate, adverse outcomes, and sentiment analysis of the feedback from the semistructured questionnaires. The completion rate was defined as the proportion of participants who attended all 3 VR intervention sessions compared to the number of participants present at the start of the first session.

Adverse outcomes refer to the number and percentage of instances recorded during the entire VR session based on VR sickness items. The VR sickness items were specially tailored to the context of our study, drawing from standard scales but modified to enhance clarity and relevance for our participants. This decision was informed by preliminary findings from a prepilot phase, where traditional VR sickness descriptors such as “fullness of the head” in the Virtual Reality Sickness Questionnaire (VRSQ) [58] were not well understood by participants. Instead, we included nine specific aspects that better matched our participants’ experiences and feedback: general discomfort, fatigue, headache, eye strain, difficulty focusing, nausea, blurred vision, dizziness, and vertigo. The severity of each symptom was rated using a 5-point Likert scale, with responses ranging from 1 (“not at all”) to 5 (“extremely”). Higher scores indicated greater symptom severity.

#### Qualitative Data Analysis

The final measure was based on the sentiment analysis of the feedback from the semistructured questionnaire. The number and percentage of positive and negative responses were categorized into 4 indicators for analysis. Participants’ feedback was analyzed using inductive thematic content analysis via Microsoft Word. No formal audit trail was kept. Reflective team discussions helped ensure coding consistency. Due to the small sample size and pilot nature, data saturation was not formally evaluated. Trustworthiness was addressed by clarifying responses during interviews and transparently describing methods, following Braun and Clarke’s [59] guidance on credibility, dependability, confirmability, and transferability.

#### Semistructured Interviews

Following the conclusive VR session, participants were invited to answer eight open-ended questions: (1) Do you enjoy the VR experience? (2) Does operating the VR equipment affect your pain level? If yes, in what way? (3) Do you find the VR system easy or difficult to operate? Please explain. (4) Are there any aspects of playing VR that you like the most? (5) Are there any aspects of playing VR that you dislike? (6) Do you prefer VR to other daily activities such as games, sports, or exercise? Please explain. (7) Would you be willing to participate in future VR experiences? (8) Do you have any other suggestions or comments about the VR experience?

### Statistical Analysis

Data analysis was performed using IBM SPSS Statistics (version 29.0). Descriptive statistics, including counts, SDs, means, and proportions, were calculated for demographic data and feasibility factors.

The Shapiro-Wilk test was used to assess data normality for outcome variables, including NPRS, Pain Self-Efficacy, WHO-5, and Mood. In addition, Les Mills BODYCOMBAT performance scores, postintervention WHO-5 scores, and average mood scores after VR sessions were evaluated separately for group analysis.

Mann-Whitney U tests and independent sample *t* tests were used to compare group differences based on data normality. Cohen *d* was calculated to assess the magnitude of VR’s impact on parametric variables. Statistical significance was set at a 2-tailed *P*<.05. Responses to the final open-ended questions were transcribed and analyzed using a combination of traditional and inductive content analysis methods, as recommended by Vaismoradi et al [60].

All hypothesis tests were exploratory; no formal adjustment for multiplicity was applied, so results should be interpreted with caution regarding potential type I error inflation.

Because multiple outcomes were analyzed without formal adjustment, *P* values near the threshold (eg, pain self-efficacy, *P*=.03) should be viewed as exploratory due to potential type I error inflation.

### Ethical Considerations

The study was conducted following the Declaration of Helsinki and approved by the Research Ethics Committee of Hong Kong Metropolitan University (HE-RD/2023/1.15). All participants provided written informed consent permitting both primary data collection and secondary analysis of deidentified data. Datasets were pseudoanonymized with numeric identifiers, and the data analyst did not have access to the reidentification key. This key was securely stored on an encrypted, password-protected device accessible only to senior members of the research team not involved in data analysis. Participants received HK$150 (ie, US $19.11) supermarket coupons as compensation for their participation. Illustrative photographs do not allow identification because VR headsets and surgical masks obscure the face; a visible coauthor granted written permission for publication.

## Results

### Participant Progression

**Figure 5.**
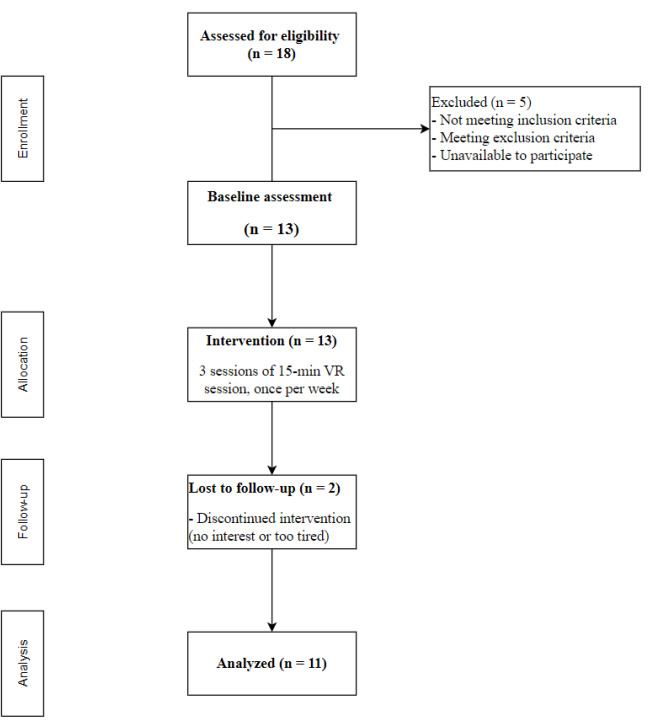
Flow diagram of participant progress throughout the intervention.

As shown in Figure 5, a total of 18 participants were recruited from the day care center. Of these, 5 were excluded based on eligibility criteria, resulting in a recruitment rate of 72.2%. During the 3 VR intervention sessions, 2 participants withdrew: 1 reported fatigue, and the other preferred not to participate further as it interfered with his usual mahjong activities. In total, 11 participants completed the intervention and were included in the final analysis.

### Demographic Characteristics

Table 1 summarizes the baseline characteristics of the 13 participants. The participants had a mean age of 79.23 (SD 9.221). Eight participants reported having chronic pain for over 3 years, while the remaining 5 were nonpainful older adults. Most of the participants had attended primary school or below, lived with others, had no VR experience, and had no glaucoma or cataract. Among the participants, 9 (69.2%) of the participants used smart devices in daily life.

**Table 1.** Baseline characteristics of the participants (N=13).

Characteristic	VR^a^ participant
Mean age (SD)	79.23 (9.221)
Sex, n (%)	
Female	7 (46.2)
Male	6 (53.8)
Education level, n (%)	
Primary school or below	11 (84.6)
Secondary school	2 (15.4)
Marital status, n (%)	
Single (widowed, divorced, or unmarried)	6 (46.2)
Married	7 (53.8)
Living arrangement, n (%)	
Living with others (spouse, children, or maid)	12 (92.3)
Alone	1 (7.7)
Employment status, n (%)	
Retired	13 (100)
Income level, n (%)	
<HK$ 5000 (ie, US $636.95)	11 (84.6)
HK$5001-HK$ 10,000 (ie, US $637.07 to US $1273.89)	1 (7.7)
HK$10,001-HK$ 20,000 (ie, US $1274.02 to US $2547.79)	1 (7.7)
Chronic pain status, n (%)	
No	5 (38.5)
Yes	8 (61.5)
Duration of pain, n (%)	
>3 y	8 (100)
With glaucoma or cataract, n (%)	
No	9 (69.2)
Yes	4 (30.8)
VR experience before, n (%)	
No	11 (84.6)
Yes	2 (15.4)
Number of smart devices used (smartphone, iPad, laptop, and smartwatches), n (%)	
Not use	4 (30.8)
In use (≤2)	9 (69.2)

a VR: virtual reality.

### Primary Outcomes

#### Preliminary Signals

Table 2 shows the efficacy of the VR intervention through a comparative analysis of scores across various outcome measures using appropriate parametric and nonparametric statistical tests.

For pain intensity measured by NPRS, despite a 20.04% decrease in the mean after the intervention, the paired samples *t* test revealed no statistically significant difference, with a small effect size (*P*=.53; Cohen *d*=−0.25).

In the case of pain self-efficacy, a median improvement of −2.0 was observed. This change was statistically significant, as shown by the Wilcoxon signed-rank test (z=−2.236; *P*=.03). No effect size was computed due to the nonparametric nature of the test.

For the WHO-5 well-being index, no increase in the median score was found, and the test indicated no statistical significance following the intervention (z=1.427; *P*=.15). The effect size was not calculated for this nonparametric test.

Finally, a significant difference was detected in mood scores. The mean score was increased by 2.64 points (16.32%), supported by a large effect size (Cohen *d*=1.82) and statistically significant (*P*<.001).

**Table 2.** Summary of primary outcomes of the virtual reality intervention.Study design: a single-arm, mixed methods pilot study. Study population: N=13 (nonpain: n=5; pain: n=8). Study time frame: virtual reality sessions were held once weekly for 3 consecutive weeks. Study location: an elderly day care center in Hong Kong.

Outcome variable	Preintervention, mean (SD)	Postintervention, mean (SD)	Preintervention, median (IQR)	Postintervention, median (IQR)	Mean difference (SD; %) or median (IQR)	z-value	*P* value	Cohen *d* (95% CI)
NPRS^a^	4.29 (2.984)	3.43 (3.69)	N/A^b^	N/A^b^	−0.86 (SD 3.44; −20.04%)	N/A^b^	.53	−0.25 (95% CI –1.00 to 0.50)
Pain self-efficacy^c^	N/A^b^	N/A^b^	3.0 (1.5‐3.0)	1.0 (1.0‐2.0)	−2.0 (−2.0 to 0.0)	−2.236	.03	N/A^b^
WHO-5^d^	N/A^b^	N/A^b^	16.0 (12-17)	16.0 (14-18)	2.0 (−2.0 to 6.0)	1.427	.15	N/A^b^
Mood^e^	16.18 (1.319)	18.82 (0.9)	N/A^b^	N/A^b^	2.64 (SD 1.45; 16.32%)	N/A^b^	<.001	1.82 (95% CI 0.86 to 2.78)

a NPRS: Numeric Pain Rating Scale.

b N/A: not applicable.

c Pain self-efficacy: a belief of chronic pain sufferers that they can perform specific activities despite pain, measured by a 5-point Likert scale from 0 (none at all) to 5 (extremely).

d WHO-5: World Health Organization–Five Well-Being Index.

e Mood: a 5-point Likert scale with 4 questions to capture the current mood states of participants.

#### Feasibility

The acceptability and applicability of the commercial-off-the-shelf VR applications by the participants were determined by three criteria:

1. Intervention completion rate: descriptively, 7 of 8 participants with pain (87.5%) and 4 of 5 without pain (80%) completed all 3 sessions.
2. Adverse outcome: the second criterion concerns VR sickness. Most participants did not have any issues with VR sickness. Out of 36 participation instances, two participants reported general discomfort, one related to the eye mask and the other to existing back pain (Table 3). In addition, one participant experienced nervousness during the game and hand fatigue afterward.
3. Sentiment analysis: the last measure of VR feasibility was based on the sentiment analysis of participants’ feedback during the conclusive interview. Positive and negative responses to 4 questions in a semistructured questionnaire were analyzed using content analysis.

**Table 3.** Summary table of adverse outcomes.

VR^a^ sickness item	Number of incidence, N=36^b^ (%)	Remark
General discomfort	2 (5.6%)	The eye mask applied a slight pressure on the participant’s nose bridge. The participant hurt his back that morning and felt uncomfortable while playing VR.
Fatigue	1 (2.8%)	The participant felt a bit nervous while playing the VR boxing game, but she felt happy and had hand fatigue after the match.
Headache	0	—
Eye strain	0	—
Difficulty focusing	0	—
Nausea	0	—
Blurred vision	0	—
Dizziness	0	—
Vertigo	0	—

a VR: virtual reality.

b The denominator is the sum of 13 participants in T1, 12 participants in T2, and 11 participants in T3. Each participant has the chance to have VR sickness, and therefore, their total number of participation is the denominator.

In Multimedia Appendix 2, 90.9% of participants (10/11) reported enjoying the VR experience. About 72.73% (8/11) indicated that VR did not induce pain, 81.82% (9/11) found VR easy to operate, and 100% (11/11) stated they would participate again. Among those with pain, approximately 27.27% mentioned a negative impact. The high percentage of positive feedback demonstrates the practicality and acceptance of VR among older adults.

#### Analysis by Pain Group

This analysis evaluates potential differences in outcomes between the pain and nonpain groups. It aims to analyze their overall well-being, immediate mood, and game performance in terms of the three outcome variables: WHO-5, mood, and score (the game score in Les Mills BODYCOMBAT) after the intervention.

As shown in Table 4, there was no significant difference between the two groups in their overall well-being and immediate mood, as measured by WHO-5 and mood scores after the intervention (*P*>.05). The lack of significance was supported by the relatively small Cohen *d* effect sizes for both variables.

For the distribution of game performance scores, the nonpain group had a median of 46,513.5 (IQR 73,674.25; range 10,676-93,648), while the pain group had a median of 8005 (IQR 39,460; range 2380-42,441). Despite this observed difference, the U value of 5 and the *P* value of .11 indicate that the difference was not statistically significant. No effect size was reported due to the nonparametric nature of the Mann-Whitney *U* test.

**Table 4.** Group analysis for pain and nonpain participants.

Variable and group		Mean (SD)	Median (IQR)	Test statistic (U)	*t* test (*df*)	*P* value	Cohen *d* (95% CI)
AT3_WHO5^a^					−0.21 (9)	.84	−0.13 (95% CI –1.36 to 1.10)
Pain		16.14 (3.08)	N/A^b^	N/A^b^	—	—	—
Nonpain		16.75 (6.65)	N/A^b^	N/A^b^	—	—	—
Ave_After_Mood^c^					0.64 (9)	.54	0.4 (95% CI –0.84 to 1.64)
Pain		18.95 (0.83)	N/A^b^	N/A^b^	—	—	—
Nonpain		18.58 (1.1)	N/A^b^	N/A^b^	—	—	—
AT3_Scores^d^					N/A^b^	.11	N/A^b^
Pain		N/A^b^	8005 (4461.5‐32089.5)	5	—	—	—
Nonpain		N/A^b^	46513.5 (17149.5‐81526)	N/A^b^	—	—	—

a AT3_WHO5: the postintervention World Health Organization–Five Well-Being Index (WHO-5) score after the third (last) virtual reality session.

b N/A: not applicable.

c Ave_After_Mood: the average postintervention mood scores.

d AT3_Scores: the scores obtained in Les Mills BODYCOMBAT after the third (last) virtual reality session.

### Qualitative Findings

In addition to the sentiment analysis, we further coded the participants’ responses into three themes. Participant feedback is detailed in Multimedia Appendix 3.

#### Theme 1: Enjoyment of VR Experience Despite Existing Conditions

Most of the participants expressed a strong appreciation for the overall VR experience, especially the 360-degree videos. These videos provided a close-to-reality feeling of traveling to places they had never been or could not visit due to varying factors such as inaccessibility, age, and physical limitations.

#### Theme 2: Physical and Psychological Impact of VR

According to the biopsychosocial model, VR and exercise had a positive influence on pain relief, as well as physical and psychological well-being [40-42]. Similar comments were found in the feedback from the participants.

#### Theme 3: Suggestions for Improvement in VR Settings and Design

Despite the overall positive feedback and the steps to adapt the nonnative commercial VR applications for older adults, one participant still raised a language barrier due to the English interface and reported comfort issues with the VR headset. Some participants also requested longer VR sessions and more varied content. These findings provide valuable insight to improve the research design and methods in the future.

## Discussion

### Principal Findings

This pilot study is one of the first to explore the feasibility and preliminary signals of nonlocalized, commercial-off-the-shelf VR applications for managing chronic pain and the well-being of community-dwelling older adults. The current study aimed to address three primary research questions: (1) evaluate the feasibility of using commercial-off-the-shelf VR applications for managing chronic pain among older adults, (2) assess the efficacy of VR in alleviating chronic pain, and (3) examine its impact on the well-being of older adults in a community setting. We found that nonlocalized commercial VR applications demonstrate good feasibility and acceptability in managing chronic pain among older adults in the community. However, their direct effects on pain intensity and overall well-being still need further exploration. These results will be discussed in detail in the following paragraphs.

### Comparison With Prior Work

First, regarding the feasibility of using commercial-off-the-shelf VR applications, VR applications have been developed specifically for chronic pain therapy or rehabilitation in clinical settings [15-22,32]. However, the high completion rates, low adverse outcomes, and positive qualitative feedback demonstrated good feasibility and acceptance of market-ready VR applications among older adults in the community. Unlike previous studies that highlighted limited English proficiency, digital literacy, and cultural relevance as challenges to VR use among older adults [36-38], our study found that a structured approach to explain key aspects to older adults and warm-up sessions before the VR intervention could effectively alleviate potential anxiety.

In addition, the practical implementation of commercial VR applications in real-world settings demonstrates considerable potential for scalability. For instance, the Meta Quest 3 is priced at approximately US $500, making it a relatively accessible technology. In contrast to other studies where VR interventions are typically administered by a qualified physiotherapist [26], a trained facilitator can also effectively oversee groups of 5-6 seated older adults after a single one-to-one orientation session. This ensures the safe and efficient use of VR applications. In addition, the option to conduct sessions at home eliminates transportation challenges, with informal caregivers able to lend support. This finding is consistent with previous studies demonstrating home-based VR therapy as a viable option for patients with chronic pain [61]. These practical factors collectively highlight the affordability, accessibility, and sustained engagement potential of commercial VR for older adults living in community settings.

Next, about the efficacy of VR for managing chronic pain and well-being, the pilot study yielded mixed results. The VR intervention showed a significant impact on emotional (immediate mood) and psychological aspects of pain management. Our qualitative findings also aligned with theoretical models [40-45], demonstrating that VR technology and exercise can provide psychological benefits. This result is consistent with the opinions of other researchers [62-64]. For instance, research showed that after receiving virtual reality hypnosis treatment, patients spent less time thinking about pain, and the reported intensity of the worst pain, anxiety, and pain discomfort were significantly reduced [63]. Distracting the participants’ attention during VR intervention may be an important psychological mechanism for pain relief [62-64]. However, no statistically significant change was detected in pain intensity or overall well-being (NPRS and WHO-5), possibly because self-efficacy might be cognitively mediated and may improve before nociceptive intensity responds [65]. This pattern aligns with contemporary pain models that emphasize affective–cognitive appraisal [66]. In small pilots, these outcomes, therefore, might not move in tandem. Larger, controlled trials with longer follow-ups are required to determine whether the psychological gains translate into clinically meaningful pain relief.

In addition, we detected no statistically significant differences in postsession mood, WHO-5 scores, or boxing-game performance between pain and nonpain participants. Although the small sample size limits broad conclusions, our results tentatively indicate that both subgroups could effectively use and enjoy the VR protocol. A larger, controlled trial is needed to confirm these findings.

### Limitations

This pilot study had several limitations that should be considered when interpreting the results. First, the study included a small number of participants in one center. The limited number of participants reduces statistical power, increasing the risk of not detecting true effects. Hence, a larger sample size from multiple centers would have provided higher statistical power for the analysis and broader applicability of the findings (refer to Multimedia Appendix 4 for the use of ChatGPT to improve the readability). For instance, based on the large mood improvement observed in this study (Cohen *d*=1.8), a two-arm trial would require approximately 34 participants per group to achieve 80% power at a 0.05 significance level.

Second, this single-arm design made it difficult to draw causal inferences about the VR intervention’s effectiveness. Because this was an uncontrolled, one-arm pilot, any pre-post change could equally be attributable to placebo effects, Hawthorne reactivity, or simple regression to the mean rather than to the VR content itself. To address these threats, we are currently planning a randomized controlled trial that will compare the same VR protocol with an attention-matched control condition.

Third, the short duration of each VR session might not have been sufficient to observe significant changes in chronic pain and well-being. Previous studies with longer and more intensive VR interventions have shown better outcomes [29]. This pilot study only collected immediate postsession outcomes; our forthcoming randomized trial will obtain 1- and 3-month follow-up data to establish the durability of effects. Future research should extend the duration and intensity of the intervention and align with physical exercise guidelines to enhance its impact. These trials should test longer interventions with a 1‐ to 3-month follow-up period to help determine if short-term psychological benefits lead to lasting reductions in pain and improvements in overall well-being.

As commercial-off-the-shelf VR applications may not fully support the local language, the research team tried to translate 360-degree videos and provided real-time interpretation for participants. However, this may still have limited participant engagement and enjoyment, as the experience was not fully immersive in their native language. While this served as a temporary solution, it highlights the need for developing native-language VR content. Future studies should prioritize VR applications in participants’ native languages whenever available (refer to Multimedia Appendix 4 for the use of ChatGPT to improve the readability). Furthermore, trials comparing language-localized and nonlocalized VR applications could show whether native-language content boosts engagement and adherence in older adults.

In addition, the reliance on self-reported measures introduced potential biases such as social desirability and recall errors. Future studies should incorporate objective measures such as the Medical Research Council (MRC) Scale for muscle strength or activities of daily living assessments to provide a more comprehensive evaluation. Performance scores from VR exercises, such as scores in Les Mills BODYCOMBAT, could also be used to objectively track progress and motivate participants, in line with self-determination theory. Future research should include objective measures of physical function, such as timed-up-and-go tests, Medical Research Council muscle grading, or assessments of daily activities, to complement self-reported outcomes.

Finally, though the participants were recruited from the same day care center, there was significant variability in their pain levels, visual abilities, and experience with VR technology. This heterogeneity could influence the results. It made it challenging to attribute outcomes solely to the VR intervention. Future research should use stratified analyses or larger samples from various locations to control for these variables. This would provide clearer insights into the intervention’s effects.

Beyond the limitations above, several practical questions also require exploration. Research in various community settings is essential to evaluate scalability. These studies should consider the current headset cost (approximately US $500) and examine the facilitator-to-participant ratio used in this study. Here, one trained staff member supported 5-6 seated users after initial training. Cost-effectiveness studies and mixed methods research on implementation would further guide real-world use. By tackling these gaps, future work can build a strong evidence base for using commercial virtual reality applications with older adults living in the community.

### Conclusions

This pilot study represents a pioneering exploration into the use of nonlocalized and commercial-off-the-shelf VR applications to manage chronic pain and well-being among older adults in community settings. This pilot demonstrated that despite language barriers, insufficient digital literacy, and cultural differences, these VR applications demonstrated good feasibility and acceptability among older adults through structured programs. Specifically, high completion rates, low adverse outcomes, and positive qualitative feedback attested to their promising prospects for application. In this uncontrolled sample, VR sessions were associated with large, within-group improvements in mood and modest gains in pain self-efficacy. While causal inferences cannot be drawn due to limitations in methodology, this study lays the groundwork for more rigorous future exploration. Through continuous research, development, and validation, VR technology is expected to become an economical, convenient, and effective nonpharmacological intervention for chronic pain management in older adults, thereby promoting aging in place and comprehensively improving their quality of life.

## Supplementary material

10.2196/67765 Multimedia Appendix 1 The scene of 360-degree videos.

10.2196/67765 Multimedia Appendix 2 Sentiment analysis of the semistructured questionnaire.

10.2196/67765 Multimedia Appendix 3 Qualitative analysis of the conclusive interview data.

10.2196/67765 Multimedia Appendix 4 An example of a ChatGPT prompt-response pair used for language editing support.

## Abbreviations

CONSORT: Consolidated Standards of Reporting Trials
GDS: Global Deterioration Scale
Mood: Mood Assessment Scale
MRC: Medical Research Council
NPRS: Numeric Pain Rating Scale
PANAS: Positive and Negative Affect Schedule
SCNAMES: Standardized Care Need Assessment Mechanism for Elderly Services
VAS: Visual Analog Scale
VR: virtual reality
VRSQ: Virtual Reality Sickness Questionnaire
WHO-5: World Health Organization–Five Well-Being Index
