# Supplementary material for: Feasibility and Efficacy of Commercial-Off-the-Shelf Virtual Reality Applications for Managing Chronic Pain and Enhancing Well-Being Among Older Adults in the Community: Mixed Methods Pilot Study
Source: JMIR Form Res. 2025 Aug 18;9:e67765. doi: 10.2196/67765 (PMC12360672; doi:10.2196/67765)
Supplement: Multimedia Appendix 1 [file formative-v9-e67765-s001.docx]

## Multimedia Appendix 1

The scene of 360-degree videos

| Theme of scenes | Website |
| --- | --- |
| Antarctica | <https://youtu.be/XPhmpfiWEEw?feature=shared> |
| Hot balloon | https://youtu.be/5nCVL_z5Doo?feature=shared |
| Volcano eruption | https://youtu.be/y9J7RUzlkz4?feature=shared |
| Polar light | https://youtu.be/cg_jfip4pSQ?feature=shared |
| Shark feeding | <https://youtu.be/oo1k24Htsa8?feature=shared> |
| Sky diving | https://youtu.be/AX4hWfyHr5g?feature=shared |
| Travelling | London: https://youtu.be/KGerjHMa90s?feature=shared  Greece: https://youtu.be/vDrNOIWm4OY?feature=shared  Sydney: https://youtu.be/5Wyb7q2p95k?feature=shared  Dubai: <https://youtu.be/ji0xO_171WA?feature=shared>  Gold Coast: <https://youtu.be/FHKXkBFmG2M?feature=shared>  Italy: <https://youtu.be/TnnRicTnrZI?feature=shared>  Washington: <https://youtu.be/I8fdxW-PwSY?feature=shared>  Jordan: <https://youtu.be/xSiv4TkfSOE?feature=shared>  Hong Kong: <https://youtu.be/KHjEUxAZ838?feature=shared>  Paris: <https://youtu.be/Cnvi6oI3Eno?feature=shared> |

| 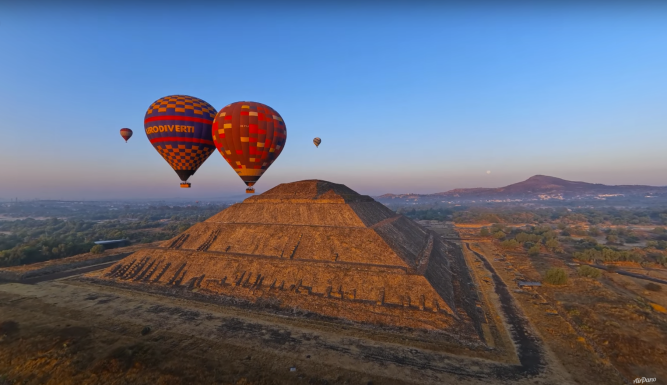 | 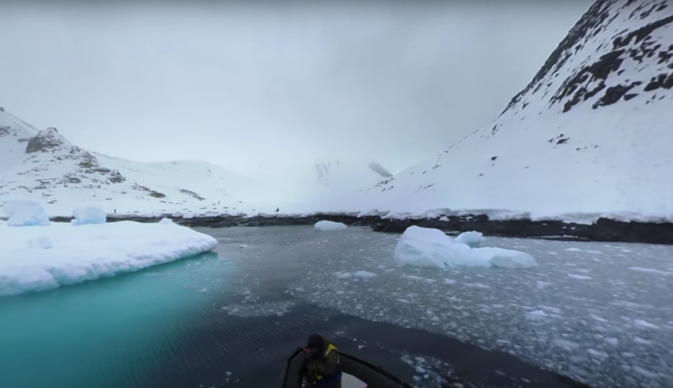 |
| --- | --- |
| 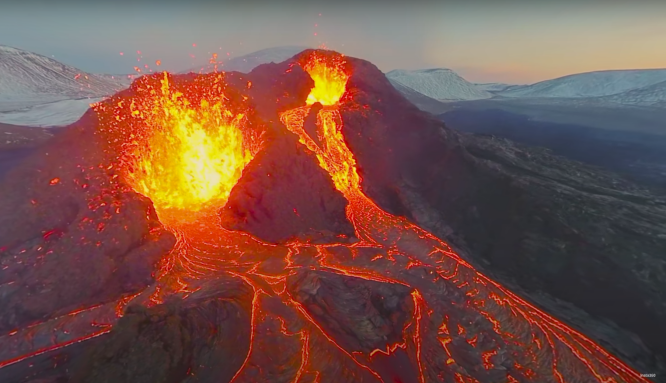 | 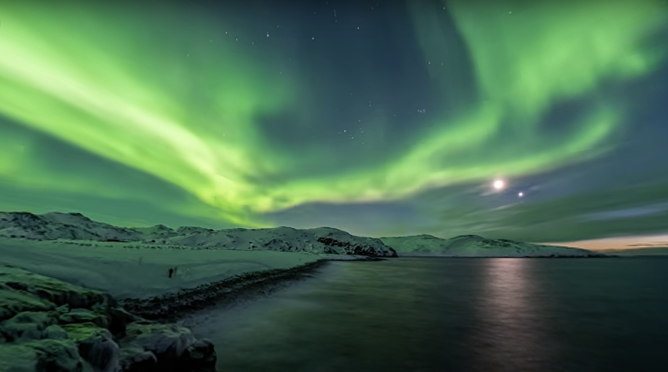 |
