# Supplementary material for: Feasibility and Efficacy of Commercial-Off-the-Shelf Virtual Reality Applications for Managing Chronic Pain and Enhancing Well-Being Among Older Adults in the Community: Mixed Methods Pilot Study
Source: JMIR Form Res. 2025 Aug 18;9:e67765. doi: 10.2196/67765 (PMC12360672; doi:10.2196/67765)
Supplement: Multimedia Appendix 2 [file formative-v9-e67765-s002.docx]

## Multimedia Appendix 2

Sentiment analysis of the semi-structured questionnaire

| Questions | Positive Responses, n (%) | Negative Responses, n (%) |
| --- | --- | --- |
| 1. “Do you like VR experiences?” | 10 (90.9%)  e.g., “I like it very much.”  “I love VR, whether it's traveling, or boxing. ” | 1 (9%)  e.g., “I don't like it, but I'll play VR when I can.” |
| 2. “Does playing VR affect pain? ” | 8 (72.73%)  e.g., “No effect, it's fun to watch.” | 3 (27.27%)  e.g., “Back pain this morning, there was pain when I played just now, but no pain when I play normally.” |
| 3. “Do you find it easy or difficult to operate a VR? ” | 9 (81.8%)  e.g., “easy.” | 2 (18.2%)  e.g., “Difficulty, cannot control, cannot read.”  “Not difficult, not really easy, average. " |
| 4. “Would you participate in the next VR experience? ” | 11 (100%)  e.g., “Yes, I will definitely attend.” | 0 (0) |
