# Supplementary material for: Feasibility and Efficacy of Commercial-Off-the-Shelf Virtual Reality Applications for Managing Chronic Pain and Enhancing Well-Being Among Older Adults in the Community: Mixed Methods Pilot Study
Source: JMIR Form Res. 2025 Aug 18;9:e67765. doi: 10.2196/67765 (PMC12360672; doi:10.2196/67765)
Supplement: Multimedia Appendix 3 [file formative-v9-e67765-s003.docx]

## Multimedia Appendix 3

Qualitative analysis of the conclusive interview data

| Theme | Comments |
| --- | --- |
| Enjoyment of VR experience despite existing conditions | - “I love using VR to explore the world, like England and other places… it allows me to see different parts of the world.” (Participant A) - “I love playing VR. I can go to places I've never been before, like deserts. I love it all.” (Participant C) - “Yes, I like to watch movies and go traveling (through VR). I don't have the opportunity to do it now. I am too old." (Participant F) - “I love using VR to travel. I can see different and inaccessible places, and I love it.” (Participant G) - “I like VR, for example, boxing, I can get some exercise, I can't really go boxing in real life. VR allows me to see places that I would never have the chance to visit in real life, such as volcanoes.” (Participant L) |
| **Physical and psychological impact of VR**  - Promoting exercise and health physically  - Feeling relaxed and excited | - “I like VR more. I like to exercise whenever I can, and moving around is good for me.” (Participant I) - “Playing VR games really lifts my spirits. I felt relaxed after playing." (Participant B) - “That's cutting-edge technology! I really enjoy boxing, the VR games. It is fun.” (Participant C) - “The game is very exciting. My heart is still beating fast after playing.” (Participant G)   Pre-intervention:   - “I'm so bored sitting around all day. It feels like I'm waiting to die. And now I'm starting to have trouble seeing things up close.” (Participant I)   Post-intervention:   - “It's so beautiful! I've seen the world and experienced a lot! I've been to a lot of places!” (Participant I) |
| Suggestions for improvement in VR settings and design | - “I can't understand the game because the language is English. Also, the VR headset is pressing down on the bridge of my nose (he wears glasses), so it would be nice if it could be adjusted more loosely.” (Participant B) - “There are so many places to visit! I want to go to Europe and see the Suez Canal. I can't get enough of VR, so I wish the experiences were longer, maybe 20-30 minutes.” (Participants C) - “Not enough time to play. It is better to play VR for half an hour.” (Participant L) |
